# Overview of microRNAs as liquid biopsy biomarkers for colorectal cancer sub-type profiling and chemoresistance

**DOI:** 10.20517/cdr.2021.62

**Published:** 2021-10-26

**Authors:** Alfred Buhagiar, Elisa Seria, Miriana Borg, Joseph Borg, Duncan Ayers

**Affiliations:** ^1^Faculty of Medicine and Surgery, University of Malta, Msida 2080, Malta.; ^2^Centre for Molecular Medicine and Biobanking, University of Malta, Msida 2080, Malta.; ^3^Faculty of medical sciences, Newcastle University, Newcastle upon Tyne NE1 7RU, UK.; ^4^Faculty of Health Sciences, University of Malta, Msida 2080, Malta.; ^5^Faculty of Biology, Medicine and Health Sciences, The University of Manchester, Manchester M13 9PL, UK.

**Keywords:** MicroRNA, miRNA, liquid biopsy, biomarkers, colorectal cancer, cancer

## Abstract

Colorectal cancer (CRC) is the third most common cancer worldwide. It has also been demonstrated that over the last ten years the incidence of CRC among younger people below the age of 50 is also increasing. Screening for colorectal cancer is of utmost importance; the rationale behind screening is to target the malignancy and reduce the incidence and mortality of the disease. Diagnostic methods to screen for incidence or relapse are therefore a requisite to detect cancer as early as possible. Scientific findings demonstrate that many deaths are due to lack of screening and therefore early identification will lead to greater survivability. In colorectal cancer, diagnostic tests include liquid biopsy biomarkers. Since the discovery of microRNAs (miRNAs), many studies have demonstrated the relationship between miRNAs and the various sub-types of CRC. Several miRNAs have been identified after analysing serum or plasma samples in patients, and such miRNAs were found to be significantly dysregulated. Such findings place the possibility of miRNAs to be at the epicentre of novel diagnostic techniques for CRC identification and sub-type stratification, including other characteristics associated with CRC development such as patient prognosis. The following review serves to underline the latest findings for miRNAs with such potential for routine diagnostic employment in CRC diagnostics and treatments.

## INTRODUCTION

Colorectal cancer is the third most common cancer worldwide. According to a conservative estimate, over 1.8 million new cases were registered globally in 2018, resulting in over 50% mortality and ranking second in terms of mortality^[1]^. In China alone, it is the fifth most prevalent disease, and since 2002 there has been an increase of 47% in its incidence^[2]^. It has also been demonstrated that over the last ten years the incidence of CRC among younger people (below the age of 50) is also increasing^[3]^. In a retrospective study, it was confirmed that CRC among the younger generation is increasing^[4]^. In the United States, an increase among young adults of CRC has been registered, and the exact reasons are still to be investigated, although the increased incidence may be attributed to external factors such as diet, obesity and lack of physical activity^[5,6]^.

A wealth of literature has been written on colorectal cancer since 1896, when Sir Jonathan Huchinson described the relationship between mucosal pigmentation and intestinal polyps, a condition that would eventually be recognised as Peutz-Jeghers syndrome^[7]^. In 1895, Dr. Aldred Warthin described Lynch syndrome as a hereditary disease^[8]^. There are various types of colorectal cancer; adenocarcinomas account for 95% of all cases, while less common types include gastrointestinal tumours (GIST) (1%), lymphomas (< 1%), carcinoids, Turcot syndrome, Peutz-Jeghers syndrome, familial colorectal cancer and juvenile polyposis coli. Estimates from the American Cancer Society reveal that 65% of CRC cases achieve survival after intervention over a five-year time period, and this survival rate can be extended if screening is continuous during this period^[9]^. Hence, success in preventing the disease long before it surfaces is of utmost importance, and detection when it is still in the early stage, with no metastases, increases the chance of survival.

## COLORECTAL CANCER SUB-TYPES

### Two major groups of CRC exist

#### The non-hereditary types are classified on the location of origin of the malignancy

(1) Adenocarcinomas originate in the lining of the internal surface of the colon. They result in the growth of polyps and if left untreated will result in cancer. They are usually removed during a routine colonoscopy^[10]^.

(2) GISTs are rare and form in cells found in the lining of the gastrointestinal tract called interstitial cells of Cajal. GISTs are classified as sarcomas since they belong to connective tissue groups, such as those of muscle, fat, deep skin tissues, nerves, blood vessels and bones^[11]^.

(3) Lymphomas: the origin of the majority of lymphomas are of the non-Hodgkins b-cell type, although other sub-types have also been identified^[12] ^and are characterised by features based on lymphoid neoplasms, according to WHO classifications^[13,14]^. Such tumours originate in localities other than lymph nodes, such as the caecum, due to the high presence of lymphoid tissues^[15]^.

(4) Carcinoids: these tumours initiate in the hormone-producing cells of the neuroendocrine origin of the enterochromaffin type, which are distributed along the gastrointestinal tract - specifically located in the crypts of lieberkuhn. Such cases are very rare, comprising less than 1% of all colonic neoplasms^[16,17]^.

#### The hereditary types of CRC are classified as follows

(1) Turcot syndrome is a variant of Lynch syndrome. It is a very rare disease; in the United States, it can register 1-2 cases per 1 million people annually^[18]^. The disease manifests itself as benign growths or polyps lining the intestinal tract and tumours to the central nervous system. It is a familial heredity disease associated with *PMS2*, *MLH1*, *APC*, *MSH2* and *MSH6* genes^[19]^.

(2) Peutz-Jeghers syndrome is a disorder that is inherited through autosomal dominance and characterised by intestinal polyps in association with skin patterns with melanin deposition^[20,21]^. Such hamartomatous polyps increase the chance of colorectal cancer^[22]^, and its incidence varies from 1 in 50,000 to 1 in 200,000 births^[23]^.

(3) Familial colorectal cancer, also known as non-syndromic^[24]^, is a group characterised by dissimilar conditions in patients with unidentified hereditary disorders and other intermittent forms that accumulate over time in families.

(4) Juvenile polyposis syndrome is a condition determined by the histology of the polyps formed in the digestive tract. The genetic condition of this syndrome can be passed from one generation to another in a family^[25]^. Based on current research, two genes are involved in this condition: *BMPR1A* and *SMAD4*^[26]^.

## SCREENING TECHNIQUES

Screening for colorectal cancer is of utmost importance. The rationale behind screening is to target the malignancy and reduce incidence and mortality of the disease. Diagnostic methods to screen for incidence or relapse are therefore a requisite to detect cancer as early as possible. Scientific findings demonstrate that many deaths are due to lack of screening and therefore early identification will lead to greater survivability. Numerous organisations such as the American Cancer society and the American College of Physicians have recommended guidelines^[27]^ for colorectal cancer screening. Such guidelines are categorised into prevention and detection. What is certain is that most of the guidelines recommend screening to start at the age of 45-50^[28]^, the age where risk starts to increase. There have been significant improvements in screening techniques and tests. In colorectal cancer, diagnostic tests are divided into three broad categories: stool-based analysis, liquid biomarkers and imaging. Evidence reveals that the use of such techniques in combination will enable the patient to improve therapeutic outcomes^[29]^.

In stool-based analysis, also known as faecal occult blood tests (FOBT), three varieties of tests can be employed which are based on guaiac oxidative reactions, immunochemical and DNA results. In guaiac-based analysis, faeces are tested for the presence of haemoglobin in the faeces. The test involves a chemical reaction by applying hydrogen peroxide through an oxidation reaction with guaiac acid via the haem molecule to convert it to guaiac blue^[30]^. The colour conversion is due to the presence of haemoglobin in the sample. Although it is affordable and non-invasive, the disadvantage is that it must be repeated at least three times, and the test can lead to false positive results due to dietary consumption by the patient before the test^[31]^. In immunochemical tests, the faeces are tested using an antibody that is specific to human haemoglobin. This test is not affected by diet, has greater specificity, requires fewer stool samples, can be used both quantitatively and qualitatively and is non-invasive^[32]^. In DNA tests, patients with CRC have DNA fragments in their stools, which have a high integrity as a marker. Such DNA fragments which originate from the exfoliation of the intestinal wall cells will have genetic abnormalities associated with K-RAS, P-53^[33]^ and epigenetic markers such as microsatellite instability^[34]^.

Imaging comes in different types and includes the following: (1) computed tomography provides either two- or three-dimensional images of the colon from magnetic resonance. Its diagnostic value is increasing with a reported sensitivity of 80.3%^[29]^. To obtain results, the bowel has to be air distended, and in some cases the patient has to ingest a contrast agent. If the images reveal any polyps, a colonoscopy follow-up must be conducted. However, at the same time, this technique has a low risk of bowel perforation and does not require sedation^[35]^. (2) Double contrast barium enema is a technique where X-ray images are obtained after the patient’s colon is covered by barium solution and then distended by air. Its use has been greatly reduced as novel imaging technology has become more available^[36]^. (3) Colon capsule endoscopy involves the swallowing of a pill-shaped camera capable of recording images as it passes through the gastrointestinal tract. It is expensive, requires uncompromising bowel preparation^[37]^ and has a reported sensitivity of 88% for polyps equal to or greater than 10 mm^[38]^. (4) Flexible sigmoidoscopy is a thin, flexible tube having a video camera at the tip which allows viewing the inside of the rectum and most of the sigmoid colon, which is the last two feet of the large intestine. Combined with FOBT tests, flexible sigmoidoscopy account for a reduction in 21% of CRC incidences and a 27% decrease in mortality^[39]^. The disadvantage is that it does not allow viewing of the entire colon as it is rather short, only about 60 cm in length. However, it does not require prolonged bowel preparation or sedation^[40]^. (5) Colonoscopy is considered as the gold standard and provides a complete image of the entire large colon and the distal part of the small colon. It also allows the ability to take a sample for biopsy during the same procedure^[41]^. The technique is expensive, requires sedation and extensive bowel preparation beforehand by the patient and there is also the risk of perforation during the procedure^[42]^.

Biomarkers: blood-based biomarkers are not new in their use. By definition, a biomarker is a biological molecule found in fluids or tissues that indicates the normal or abnormal conditions of the patient. Such serological biomarkers include carcinoembryonic antigen, epigenetic changes, DNA, RNA and microRNA. In cancer, gene expression alters significantly during the early stage of carcinogenesis. Studies have demonstrated that various genes are notably expressed in CRC, including spondin-2^[43]^, Trail-R2^[44]^ and BCNP1^[45]^.

Epigenetic modifications are also registered during carcinogenesis. Epigenetic changes are heritable alterations that influence certain genes but do not alter DNA sequence. Basically, such alterations occur as: (1) DNA methylation^[46]^, where it was elucidated that LINE-1 retrotransposon, if hypomethylated, is associated with unfavourable prognosis in CRC patients; (2) histone modifications, where deacetylation of histones affect the activity of *CDX2* gene^[47]^; and (3) changes in microRNA expression profiles, which, due to their high tissue specificity, stability and altered expression, can give a clear picture of the malignancy in terms of prognostic and diagnostic markers^[48]^.

## CLINICAL IMPORTANCE OF LIQUID BIOPSIES

In recent years, one of the major efforts focused upon by biomarker researchers and clinicians alike involves the quest for novel biomarkers that can be evaluated within the clinical setting, utilising the least-invasive procedures as possible on the patient and concomitantly requiring non-complex and rapid analytical methodologies. However, the reliability and robustness of such novel biomarkers should not be reduced, despite being collected from the individual patient in a quick and painless manner.

Consequently, the concept and eventual emergence of the research niche concerning liquid biopsies was developed at the turn of the 21st century. In essence, liquid biopsies are based on analytical methodologies focusing on reliable biomarkers that are present solely within human bodily fluids, including blood, urine, sweat, semen and saliva. In this manner, samples are collected without the requirement for highly invasive and discomforting tissue biopsies from the individual patient. In addition, also in part due to the ease of sample collection, such biomarkers can be utilised for diagnoses of early, asymptomatic phases for a particular disease condition. Presently, most of the established liquid biopsy analytical tests or screens focus on blood (whole, plasma or serum based) samples within the clinical settings, mainly due to blood being a formidable vehicle for an abundance of varying potential biomarkers (of differing origins), albeit there exists a trade-off since blood sampling can be discomforting for a small segment of the patient population due to needle-stick invasiveness of the sampling procedure.

In this respect, microRNAs (miRNAs) are highly adept for acting as liquid biopsy-based biomarkers as they can be found, apart from within cellular cytoplasmic regions, in almost all bodily fluids and within the bloodstream (as free-circulating miRNAs or transported within exosomes). In addition, the body of evidence regarding the robustness of miRNAs as reliable diagnostic and prognostic biomarkers is clearly established. Furthermore, analytical techniques for assessing miRNA biomarker-based expression profiles can be easily and rapidly performed within the clinical laboratory setting through the employment of RT-qPCR techniques. The following sections serve to describe the possibilities offered by miRNAs to serve as CRC biomarkers, particularly regarding the influence of miRNAs in CRC chemoresistance properties.

## DYSREGULATED MIRNAS IN COLORECTAL CANCER

miRNAs are small non-coding RNAs having 20-22 nucleotides, and they have a major role in affecting protein expression, much needed for cell differentiation, growth and development, and are involved in the regulation of cell function and gene regulation through translational repression, gene silencing and eventual decay of mRNA^[49]^. Since the discovery of miRNAs, many studies have demonstrated the relationship between miRNAs and the various sub-types of CRC (see Table 1). Several miRNAs have been identified after analysing serum or plasma samples in patients and were found to be significantly dysregulated.

**Table 1:** Dysregulated miRNAs in CRC

**Condition**	**Dysregulated miRNAs**		**Ref.**
	**Upregulated**	**Downregulated**	
Carcinoids	miR-183, miR-488, miR-19a, miR-19b	miR-133a, miR-145, miR-146, miR-222, miR-10b	[50]
	miR-885-5p		[51]
	miR-96, miR-182, miR-183, miR-196a miR-200a	miR-31, miR-129-5p, miR-133a, miR-215	[52]
GIST	miR-494	miR-218 miR-21	[53] [54] [55]
		miR-148b-3p miR-186 miR-221, miR-222 miR-133b	[56] [57] [58] [59]
		miR-137 miR-518a-5p miR-152	[60] [61] [62]
Adenocarcinoma	miR-17, miR-92a, miR-20a, miR-19b, miR-18a, miR-21 miR-196b	miR-145 miR-4299	[63] [64]
	miR-141, miR-19a, miR-20a, miR-19b-1, miR-19b-2, miR-16, miR-590, miR-335		[65]
	miR-182 miR-135b		[66] [67]
	miR-503, miR-4417, miR-18a, miR-431, miR-1246, miR-18b	miR-375, miR-378, miR-139-5p, miR-133a, miR-422a	[68]
Lymphomas	miR-21 miR-338-5p miR-155-5p, miR-200c-3p, miR-130a-3p, mir125b-5p, miR-130a	miR-451 miR-451a, miR-145-5p	[69] [70] [71]
	miR-494, miR-21	miR-28	[72] [73]

They act as tumour inducers, an oncomiR, by targeting tumour suppressor genes and promoting oncogenesis by downregulating such tumour suppressors^[74]^. This aberrant expression has been acknowledged to influence signalling pathways, including WNT/β-catenin, epidermal growth factor receptor, transforming growth factor-beta and epithelial-to-mesenchymal transition pathways^[75]^. Dysregulated miRNAs have been attributed to the initiation, progression and metastasis of CRC. They have also been credited for chemoresistance exhibited in various tumours including gastric, lung and breast. For example, in CRC, miR-223 in conjunction with FBXW7 pathway was demonstrated to increase chemoresistance to doxorubicin^[76]^. They also cause resistance to radiotherapy, as demonstrated by miR-630, which triggers a protein kinase TP53RK that causes cancer cells to become resistant. In addition, miR-62 confers resistance to radiotherapy by activating the apoptosis gene in cancer cells^[77]^.

By no means is this list exhaustive; a search on PubMed has identified comprehensive reports listing many dysregulated miRNAs^[78-80]^. Meanwhile, in the CRC hereditary types, this research niche is still in its infancy and lacks any identifiable dysregulated miRNAs, and consequently scientific literature on this research theme is very limited.

## MICRORNAS AS BIOMARKERS IN COLORECTAL CANCER

The use of circulating miRNAs as diagnostic biomarkers is very promising. Studies have persistently demonstrated that miRNAs have a particular signature for every cancer sub-type such as breast, prostate, lung and colorectal. They are very stable in the body and have been identified in saliva, urine, plasma, serum, tears and breast milk^[81]^. Their stability is attributed to their packaging in micro-vesicles and exosomes and because they are bound to the argonaute proteins^[82]^, which protect them against degradation.

**Figure 1:**
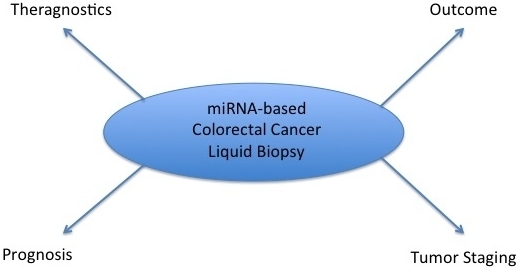
Outline of miRNA-based CRC biomarker potential clinical application.

In a meta-analysis literature review of 34 scientific papers, it was elucidated that tests on serum or plasma gave a solid result of 76% sensitivity and specificity in terms of a single dysregulated miRNA signature. In all of these studies, 28 miRNAs were identified as potential biomarkers. These results demonstrate the capability of miRNA to be used as a diagnostic and prognostic tool for the detection of CRC (see Figure 1)^[83]^.

The use of miRNA in liquid biopsies is very advantageous and promising. Its principal advantage is the fact that tests are less invasive than colonoscopy, which despite being the gold standard tends to be discomforting. This minimal invasiveness in biopsy allows a wide-ranging analysis of protein levels, tumour stage, treatment effectiveness^[84]^ and chemoresistance - all associated with the tumour in question. The idea of using a panel of miRNAs as a diagnostic tool has gained substantive ground. A literature review demonstrated that a combination of several miRNAs^[85]^ can have a sensitivity of 84.7% and a specificity of 98.7% as compared to other established biomarkers. In gastric cancer, a similar study demonstrated that a panel of six miRNA (combining miR-18a, miR-19a, miR-21, miR-92a, miR-199a and miR-421) is very^[86]^ effective as a diagnostic assessment.

Circulating miRNAs are very effective biomarkers to indicate different sub-types of cancer; they can also be used to monitor tumour metastasis and progression, indicate sensitivity to clinical treatment, have a high prognostic value in the disease and present a high sensitivity^[87]^. Their profiles also change at different stages of the malignancy, as significantly elucidated in a study, which demonstrated that the miRNA profile changed at every stage of CRC formation^[88]^. Several miRNA panels have been validated as biomarkers for CRC, as indicated in Table 2.

**Table 2:** miRNA profiles in CRC

**miRNA panel**	**Biomarker viable use**	**Ref.**
miR-23a-3p, miR-27a-3p, miR-142-5p, miR-376c-3p	Diagnostic and prognostic	[89]
miR-15b, miR-215, miR-145, miR-192, let-7g	Prognostic	[90]
Let-7a, miR-1229, miR-1246, miR-150, miR-21, miR-223, mir23a	Diagnostic	[91]
miR-143-5p, miR-27a-3p, miR-31-5p, miR-181a-5p, miR-30b-5p, miR-30d-5p, miR-146a-5p, miR-23a-3p, miR-150-5p, miR-210-3p, miR-25-3p, miR-196a-5p, miR-148a-3p, miR-222-3p, miR-30c-5p and miR-223-3p	Prognostic (stage II and III)	[92]
Let-7i, miR-10b, miR-30b	Prognostic (high risk stage II)	[93]
miR-5010-3p, miR-5100, miR-656-3p, miR-671-3p	Prognostic (stage II)	[94]
miR-19a-3p, miR-21-5p, miR-425-5p	Diagnostic	[95]
miR-103a-3p, miR-127-3p, miR-151a-5p, miR-17-5p, miR-181a-5p, miR-18a-5p, miR-18b-5p	Diagnostic	[96]
miR‐21, miR‐25, miR‐18a, miR‐22	Diagnostic	[97]
miR-301a, miR-23a	Diagnostic	[98]
miR-1290, miR-320d	Diagnostic	[99]
miR-20a, miR-486	Diagnostic	[100]
miR-223, miR-92a	Diagnostic	[101]

The demonstrated stability in liquid biopsies with a high degree of specificity and sensitivity indicates their effectiveness as a non-invasive and low-cost test; however, they do come with limitations. One of the problems is the standardisation of tests and origin of samples. In one study, it was identified that miRNAs in arterial plasma were higher in concentration than in venous plasma^[102]^. It was also discovered that a panel of miRNAs was found to be significantly lower in peripheral vein and tumour tissue than in mesenteric veins in colorectal cancer.

This suggests that the panel signature is more complete in the tissue rather than in the veins. This highlights the problems encountered in sampling, which should be carefully considered before doing the tests^[103]^. Another factor is the difficulty of using single miRNA to distinguish between malignant and benign tumours. miR-21-5p cannot distinguish benign polyps from carcinoma in CRC patients^[104]^.

## MIRNA BIOMARKERS FOR CRC CHEMORESISTANCE

Non-coding RNAs, including the miRNA family, have undoubtedly proven to be of ubiquitous importance across all physiological realms, including CRC chemoresistance^[105]^.

The study conducted by Sasaki *et al*.^[106]^ just recently highlighted the importance of plasma miR-33a-5p to act as an effective biomarker for foresight on metastatic CRC chemotherapeutic success, focusing specifically on the typical first-line treatment combination of chemotherapeutic drugs, namely fluoropyrimidine + oxaliplatin + bevacizumab. The results of this study reveal that non-responders to CRC chemotherapeutic cycles had upregulated miR-33a-5p, indicating that patients with downregulated plasma levels for this miRNA would be more likely to respond to CRC chemotherapy^[106]^.

Another study conducted earlier in 2021 by Li *et al*.^[107]^ demonstrated that miR-490-3p has the capacity to thwart migration properties and chemoresistance traits (against cisplatin and fluorouracil) in CRC cells, through controlling TNKS2. Following CRC cell line-based miRNA functional analysis studies, miR-430-3p was found to be downregulated in CRC cells, and it was negatively associated with migrative properties in CRC and the corresponding cellular invasiveness^[107]^.

Furthermore, a study conducted earlier in 2021 by Mou *et al*.^[108]^ also revealed that miR-1254 has regulatory influences on oxaliplatin resistance within human CRC cell lines.

## CONCLUSION

Other factors might influence the true profile of miRNAs, including gender, age, ethnicity and disease history. Therefore, additional studies are of paramount importance to validate the efficacy of using circulating miRNAs as biomarkers. Results from larger cohorts could identify reliable data and increase their potential as a non-invasive biomarker, to serve as an important clinical application in early diagnosis and to provide prognostic value in CRC.

## DECLARATIONS

### Copyright

© The Author(s) 2021.
